# Disease duration modulates BOLD representations of alpha oscillations in drug‐resistant epilepsy: A concurrent EEG and fMRI study

**DOI:** 10.1111/epi.18685

**Published:** 2025-11-15

**Authors:** Jia‐Hong Sie, Hsin‐Ju Lee, Yen‐Cheng Shih, Chien‐Chen Chou, Chien Chen, David Niddam, Hsiang‐Yu Yu, Fa‐Hsuan Lin, Wen‐Jui Kuo

**Affiliations:** ^1^ Institute of Neuroscience National Yang Ming Chiao Tung University Taipei Taiwan; ^2^ Physical Sciences Platform Sunnybrook Research Institute Toronto Ontario Canada; ^3^ Department of Medical Biophysics University of Toronto Toronto Ontario Canada; ^4^ Department of Neurology, Neurological Institute Taipei Veterans General Hospital Taipei Taiwan; ^5^ Brain Research Center National Yang Ming Chiao Tung University Taipei Taiwan

**Keywords:** alpha oscillations, concurrent EEG and fMRI, disease duration, drug‐resistant epilepsy

## Abstract

**Objective:**

Alpha oscillations play a fundamental role in top‐down cognitive processes by regulating cortical excitability. Building on previous reports of altered alpha activity in patients with drug‐resistant epilepsy (DRE), we conducted concurrent electroencephalography (EEG) and functional magnetic resonance imaging (fMRI) studies to investigate the blood oxygen level–dependent (BOLD) correlates of alpha oscillations and assess whether these responses are modulated by disease duration.

**Methods:**

Twenty‐seven adults with focal DRE were recruited to undergo concurrent EEG and fMRI studies. Alpha oscillations were defined as the frequency range spanning from 7 Hz to the individual alpha peak frequency (IAPF) plus 2 Hz. Both relative alpha power fluctuations and epilepsy duration were included in the fMRI data analysis.

**Results:**

The BOLD responses associated with alpha oscillations in patients with DRE differed from those previously reported in healthy individuals. Epilepsy duration was negatively correlated with IAPF and modulated alpha‐related activity in brain regions implicated in sympathetic regulation: (1) increased activation in the paracentral lobule and right anterior and posterior insular cortices, and (2) decreased activation in the bilateral angular gyri and left orbitofrontal gyrus.

**Significance:**

These findings advance our understanding of how epilepsy interacts with the central autonomic system by revealing distinct modulation of alpha activity. Although brain imaging is critical in epilepsy assessment, MRI findings in patients with DRE are often unremarkable. This study provides an alternative approach to characterizing disease progression in DRE.

**Key points**

- Concurrent electroencephalography (EEG) and functional magnetic resonance imaging (fMRI) was used to investigate neuronal effects of epilepsy duration through the individual alpha peak frequency (IAPF) in patients with drug‐resistant epilepsy (DRE).
  IAPF correlated negatively with epilepsy duration in DRE patients.
- The relative IAPF power fluctuations positively correlated with blood oxygen level–dependent data in the orbitofrontal cortex, thalamus, and cerebellum.
- Epilepsy duration modulated alpha‐related activity in brain regions implicated in sympathetic regulation.
- This study provides an alternative approach to characterizing disease progression in DRE.

## 1 INTRODUCTION

Prolonged uncontrolled seizures, such as in drug‐resistant epilepsy (DRE), have profound consequences, including inadequate attention, language, memory, and globally decreased cognition, ultimately diminishing overall quality of life.^1^, ^2^ DRE has been defined as the failure of two or more adequate trials of antiseizure medication, due to inefficacy and not intolerance, to achieve seizure freedom.^3^, ^4^, ^5^ Community‐based data have indicated that up to 22.5% of patients with epilepsy have DRE.^6^ The mechanisms underlying drug resistance are complex and multifactorial.^7^ Experimental animal studies demonstrate that repeated seizures induce progressive and cumulative changes in neural circuits. However, in human epilepsy, our understanding of seizure‐induced alterations of cortical representations in relation to seizure type, duration, or frequency remains limited.^8^ In this study, we focused on epilepsy duration as a critical factor for epilepsy progression in DRE.

Alpha oscillations^9^ have been recognized as the most salient electroencephalography (EEG) components and are fundamental to top‐down cognitive processes by governing cortical excitability.^10^, ^11^, ^12^, ^13^, ^14^, ^15^, ^16^, ^17^ Alpha oscillations have been implicated in attention,^18^ perception,^19^, ^20^ functional inhibition,^21^ working memory,^22^ and cognitive control.^14^, ^15^, ^16^ In patients with DRE who experience recurring seizures, brain circuits may undergo progressive remodeling, which may escalate the severity of epilepsy and impact cognition and behaviors. EEG research of epilepsy classification has revealed alterations in alpha oscillation, characterized by the changes in both frequency and topography.^23^, ^24^ Recent research further highlights a significant trend of a slowed and frontally spreading alpha rhythm in heterogeneous epilepsy cohorts.^25^ In addition, normalized spectral power of alpha activity may help in identifying patients with epilepsy, even in the absence of epileptiform activity, recorded seizures, or other EEG abnormalities.^26^

Note that, given individual differences, the individual alpha peak frequency (IAPF) has been used increasingly to assess intra‐individual variability in the alpha rhythm, particularly in relation to healthy aging, hormonal changes, and cognitive traits.^27^, ^28^, ^29^ Reducing or increasing IAPF would result in changes of the individual's performance level.^30^, ^31^, ^32^ Therefore, building on this evidence, alpha oscillations, particularly the IAPF, can offer a lens through which to examine the neuronal effects of disease duration in DRE patients.

In this study, we conducted concurrent EEG and fMRI recording on patients with DRE in a resting‐state setting. This setting can induce dynamic power fluctuations across a broad range of brain oscillations.^33^ By correlating spontaneous power fluctuations of EEG oscillations with concurrently recorded functional magnetic resonance imaging (fMRI) signals, previous studies have identified a selective set of alpha‐related brain regions.^34^, ^35^ Specifically, the global field power of alpha oscillations has been shown to correlate positively with activity in the cingulo‐opercular/insular network but negatively with activity in the dorsal parietofrontal network.^36^ In addition, long‐range phase synchrony of alpha oscillations correlates positively with activity in the frontoparietal network.^37^ Based on this evidence, we hypothesized that disease duration would not only correlate with the patients' IAPF but also influence the regional blood oxygen level–dependent (BOLD) dependency of IAPF power fluctuations within these networks.

## 2 METHODS

In general, we identified the IAPF and extracted the spectral power fraction within the range of 7 to IAPF+2 Hz, within the broader 1–14 Hz band, to track its fluctuations during MR scanning. The fluctuations were then convolved with the hemodynamic response function (HRF) for fMRI data modeling to map their spatial distribution in the brain. We also included disease duration as a covariate in the fMRI data analysis, to assess its influence on regional BOLD responses associated with alpha oscillations of the patients. Details are elaborated in the following sections.

### 2.1 Participants

The study protocol was approved by the Taipei Veterans General Hospital Institutional Review Board, and the participants provided written informed consent before scanning. Twenty‐seven adult patients with DRE were recruited for participation in this study. Clinical diagnosis and classification were performed according to the International League Against Epilepsy (ILAE) 2025 classification of epilepsies and seizures.^3^ All patients were diagnosed with focal epilepsy. Specifically, 12 patients (44%) had temporal lobe epilepsy and 15 patients (56%) had extra‐temporal epilepsy. The patients who underwent surgery or were classified as generalized seizure cases were excluded. Table 1 presents the demographic and clinical characteristics of the patients.

**TABLE 1:** Demographic characteristics of the patients (*n* = 27).

Characteristics	Mean ± SD
Age, years	33.1 ± 9.0
Sex, female/male	11/16
Education, years	14.2 ± 2.4
Onset age, years	17.5 ± 7.9
Duration, years	15.6 ± 7.2
Full‐scale IQ	87.4 ± 12.6
IAPF (Hz)	8.8 ± 1.2

*Note*: The values are illustrated as mean ± SD.

Abbreviations: IAPF, individual alpha peak frequency; IQ, intelligence quotient; SD, standard deviation.

### 2.2 Simultaneous EEG and fMRI recording

Data acquisition in this study was conducted in a resting‐state condition, during which the participants were encouraged to relax with their eyes closed. EEG data were acquired with a 32‐channel MR compatible EEG system (BrainCap MR, BrainVision Recorder Version 1.21). All electrodes in the cap had sintered Ag/AgCl sensors incorporating 10 kΩ safety resistors (5 kΩ: tip +5 kΩ: box). The separate electrocardiography electrode had a built‐in 20 kΩ resistor (15 kΩ: tip +5 kΩ: box). The EEG sampling rate was set at 5 kHz. The electrode impedances and the impedances of the reference and ground electrodes were set below 9 kΩ.

fMRI data were acquired with a 3 T MRI system (Siemens, Erlangen, Germany) and with a 64‐channel head coil. The structural T1 image was acquired with the magnetization‐prepared rapid gradient echo (MPRAGE) pulse sequence in the sagittal plane (repetition time [TR] = 2530 ms, echo time [TE] = 3.3 ms, resolution = 1 × 1 × 1 mm^3^, field of view [FOV] = 256 mm, flip angle = 7°, slice numbers = 192, matrix size = 256 × 256, generalized auto‐calibrating partial parallel acquisition (GRAPPA) acceleration factor = 2). Data from 11 patients were acquired with the Skyra 3 T system. Functional images were acquired with a T2*‐weighted echo‐planar imaging (EPI) sequence (TR/TE = 1980/26 ms, FOV = 256 mm, flip angle = 90°, slice numbers = 33, resolution = 4 × 4 × 4 mm^3^, GRAPPA acceleration = 2). Data were acquired from 16 patients with the Prisma 3 T system. Functional images were acquired with a T2*‐weighted EPI sequence (TR/TE = 2000/26 ms, FOV = 256 mm, flip angle = 90°, slice numbers = 40, resolution = 4 × 4 × 4 mm^3^, GRAPPA acceleration = 2). There were 240 (TR) repetitions for a session of fMRI data.

For data acquisition, two EEG sessions were acquired right after EEG setup in the preparation room. There were four sessions of concurrent EEG and fMRI recordings. Each session took 8 min. The participants were asked to close their eyes during data acquisition.

### 2.3 EEG data processing

EEG data processing was conducted with the BrainVision Analyzer 2.2.2 software (Brain Products, GmbH, Gilching, Germany), which included (1) MR‐gradient artifact removal^38^; (2) down‐sampling to 250 Hz; (3) band‐pass filter between 1 and 30 Hz; (4) robust QRS complex detection in the fMRI of the brain (FMRIB) plug‐in for EEGLAB, followed by a correction based on the subtraction of an averaged artifact template^39^; (5) removal of the first 6 s data; and (6) further application of the FastICA package to the concatenated data to remove artifact components for obvious eye movement, cryogenic pump operations, and residual magnetic field gradients. For the two sessions of EEG recorded without MR scanning artifacts, the EEG data were processed with Steps 2, 3, 5, and 6.

### 2.4 MR image processing

Image data processing included slice timing correction, outlier detection, direct segmentation and Montreal Neurological Institute (MNI)–space normalization, and smoothing with CONN toolbox.^40^ Functional and anatomic images were normalized into standard MNI space; segmented into gray matter, white matter, and cerebrospinal fluid (CSF) tissue classes, and resampled to the original functional resolution using statistical parametric mapping statistical parametric mapping (SPM) unified segmentation and normalization procedure. Functional images were smoothed using spatial convolution with a Gaussian kernel of 8‐mm full‐ width at half maximum (FWHM). Finally, functional data were denoised by including the regressors of potential confounding effects characterized by white matter time series (5 CompCor noise components), CSF time series (5 CompCor noise components),^41^ motion parameters and their first‐order derivatives (12 factors), liner trends (2 factors), and physiological noise time series from peripheral measures (14 factors for physiological noise via retrospective image‐based correction [RETROICOR]^42^ using Fourier expansions for the estimated phases of cardiac pulsation [second order], respiration [second order], and cardiorespiratory interactions (first order), respiratory volume per time [RVT],^43^ and heart rate variability [HRV]^44^ by using MATLAB PhysIO toolbox^45^) within each scanning session, followed by band‐pass frequency filtering of the BOLD time series between .008 Hz and .09 Hz.

### 2.5 Relative alpha power fluctuations

**FIGURE 1:**
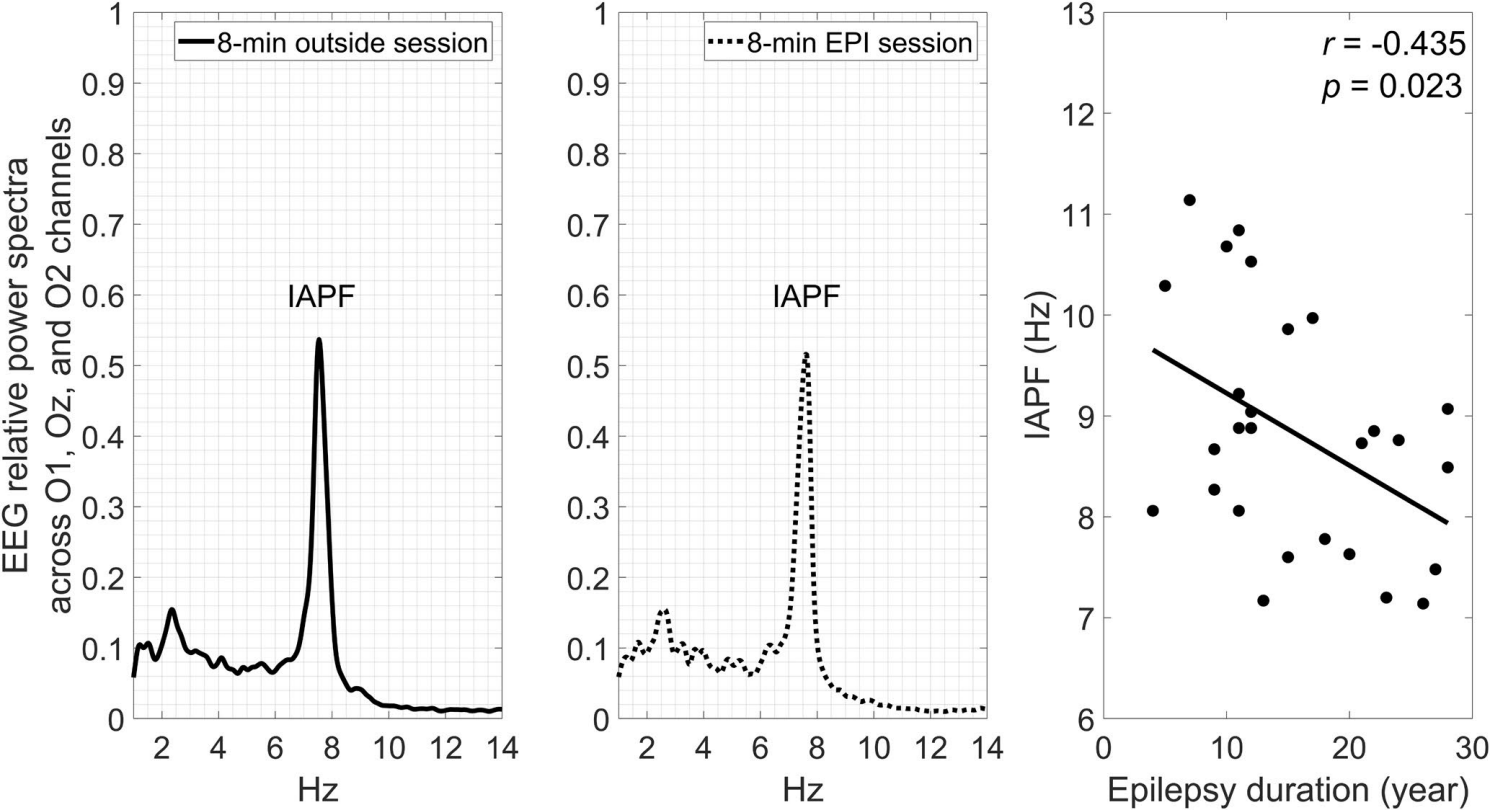
Electroencephalography (EEG) relative power spectra across O1, Oz, and O2 channels in an EEG‐only session (left panel) and a concurrent recording session (middle panel) from a representative patient. Both sessions show individual alpha peak frequency (IAPF) of about 7.63 Hz. The right panel shows negative correlation between the patients' IAPF and epilepsy duration (*r* = −0.435; *p* = 0.023).

The IAPF is the maximum power frequency within the EEG frequency band of 7–14 Hz. To determine the IAPF based on the dominant amplitude of the EEG, we analyzed data from an external session, extracting the spectral alpha peak frequency between 1 and 14 Hz from the O1, Oz, and O2 channels, as shown in the left panel of Figure 1 from a female patient. The middle panel of Figure 1 illustrates that the IAPF was preserved in the preprocessed EEG data during an 8 min EPI scan of the same patient. The IAPFs from both sessions were fully consistent with each other.

To derive the relative alpha power fluctuation, a spectrogram was first generated across four concurrent sessions using a short‐term Fourier transform with a four‐term Blackman‐Harris window for each EEG channel. The window length was 2 TR and moved with a step size of 1 TR, resulting in an overlap of 1 TR between consecutive windows. The temporal resolution of the spectrogram matched the TR of the BOLD resting‐state scans, yielding spectrograms with dimensions of 31 channels × 240 windows (with a window length of 2 TR).

In each spectrogram, the relative alpha power (7 to IAPF+2 Hz) was computed by normalizing it to the total power within the 1–14 Hz analysis band. Finally, the relative alpha power fluctuation was obtained by calculating the root mean square across the O1, O2, and Oz covering the occipital region and convolving the result with a canonical HRF. This fluctuation was then used as a regressor for fMRI data analysis.

### 2.6 EEG‐informed fMRI statistical analyses

After MR image preprocessing, the confound‐corrected scans were analyzed using a general linear model, incorporating a regressor for relative alpha power fluctuation and a nuisance regressor accounting for slow signal drift below 1/128 Hz across different sessions. This analysis was conducted using SPM12 (https://www.fil.ion.ucl.ac.uk/spm/).

Statistical results were estimated for voxels across the whole brain mask. The resulting maps of relative alpha power fluctuations across sessions were used for further group analyses in a regression model. Epilepsy duration and scanner type were included as covariates. Statistical parametric maps were thresholded at *p* = .005, with a cluster‐wise false discovery rate (FDR) correction at *p* = .05 for multiple comparisons.

## 3 RESULTS

At the behavioral level, first, the full‐scale intelligence quotient (IQ) was lower than the mean (100 ± 15) of the norm (*z* = −4.38; *p* < .001). Second, the IAPF (8.83 ± 1.18 Hz) was significantly lower than 10 Hz (*t* = −5.17; *p* < .001). Finally, the patients' IAPF was negatively correlated with epilepsy duration (*r* = −.435; *p* = .023) (the right panel of Figure 1).

**FIGURE 2:**
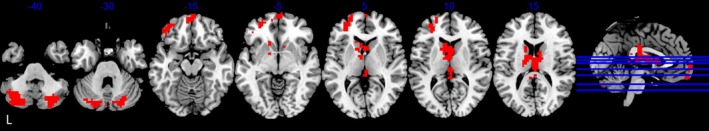
Positive bold oxygen level–dependent (BOLD) correlates of relative alpha power fluctuation. Results were thresholded at voxel *p* = .005 and corrected at cluster level with a false discovery rate (FDR) *p* = .05. L, left.

For the IAPF‐related brain activity, the fMRI data indicated that BOLD signals in subcortical regions, including the thalamus, caudate, and bilateral cerebellar cortices, as well as in cortical regions, including the left ventral and medial prefrontal cortices, fluctuated in phase with the IAPF power fluctuations, as shown in Figure 2 (see Table 2). No brain area showed BOLD signals negatively correlated with the IAPF power fluctuations.

**TABLE 2:** Summary of BOLD correlates associated with relative alpha power fluctuation.

Brain area	BA	Cluster size (voxels)	Max (T)	MNI (mm)		
				*x*	*y*	*z*
Positive correlates						
Thalamus/caudate		244	5.30	2	−22	12
Left cerebellum		123	4.92	−42	−62	−40
Right cerebellum		100	4.63	22	−78	−36
Left prefrontal cortex	47/10/11	90	4.50	−30	50	−12
Medial prefrontal cortex	10/11	43	4.08	2	66	0

*Note*: Results were thresholded at *p* = .005 (uncorrected) for voxels and *p* = .05 (FDR corrected) for clusters.

Abbreviation: BA, Brodmann area; BOLD, blood oxygen level–dependent; FDR, false discover rate; MNI, Montreal Neurological Institute.

**FIGURE 3:**
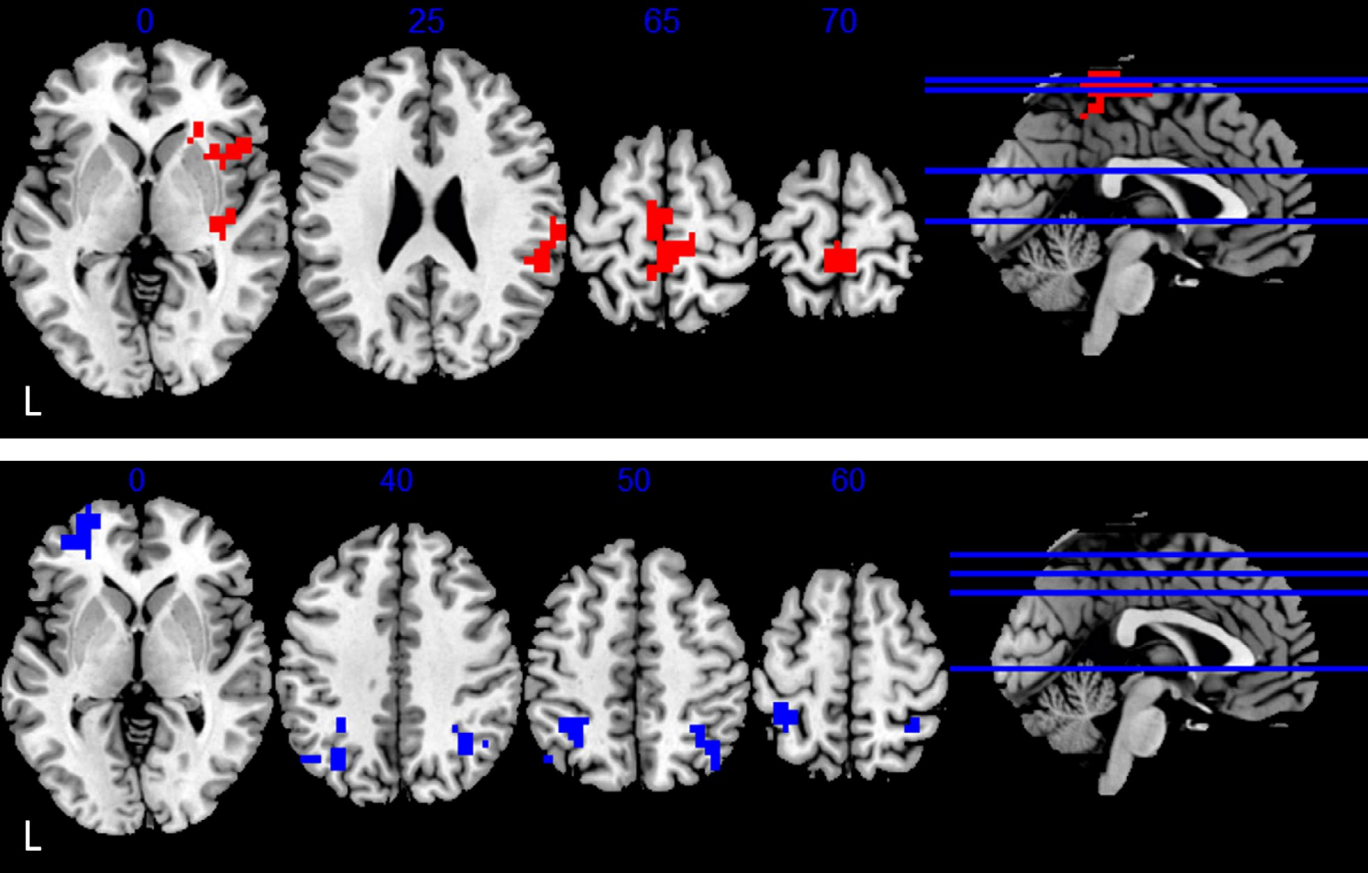
Up‐ (top) and down‐ (bottom) regulation of epilepsy duration. Results were thresholded at a voxel *p* = .005 and corrected at cluster level with a false discover rate (FDR) *p* = .05. L, left.

Regarding the effects of disease duration, we found, first, that it upregulated the IAPF‐related activity in the paracentral lobule, right anterior insula, and right posterior insula/ Supermarginal gyrus (SMG), as shown in the top panel of Figure 3 (see Table 3A). Second, it downregulated the IAPF‐related activity in the bilateral angular gyrus and left orbitofrontal gyrus, as shown in the bottom panel of Figure 3 (see Table 3B).

**TABLE 3:** Summary of modulation of epilepsy duration.

Brain area	BA	Cluster size (voxels)	Max (T)	MNI (mm)		
				*x*	*y*	*z*
(A) Upregulation						
Paracentral lobule	4	127	4.52	2	−42	68
Right posterior insula/SMG	48	81	4.55	54	−14	12
Right anterior insula	48	73	4.10	22	26	4
(B) Downregulation						
Left angular gyrus	39	87	−4.38	−34	−66	40
Left orbitofrontal gyrus	11/47	86	−4.50	−26	62	0
Right angular gyrus	39	55	−3.50	50	−58	−36

*Note*: Results were thresholded at *p* = .005 (uncorrected) for voxels and *p* = .05 (FDR corrected) for clusters.

Abbreviation: BA, Brodmann area; FDR, false discovery rate; MNI, Montreal Neurological Institute; SMG, Supermarginal gyrus.

## 4 DISCUSSION

This study investigated how epilepsy duration shapes brain activities associated with alpha oscillations in patients with DRE. Three aspects of the results, including behavioral performance, IAPF‐related BOLD responses, and the regulation effects of disease duration, were discussed.

### 4.1 Cognitive decline

At the behavioral level, we observed that the patients with DRE exhibited lower cognitive ability, as indicated by a lower full‐scale IQ score (<100) and a slower IAPF (<10 Hz). This cognitive decline may be attributed to epilepsy, as IAPF was negatively correlated with disease duration—the longer the duration, the slower the IAPF. This is consistent not only with behavioral findings from previous studies,^1^, ^2^ but also with observed neurophysiological alterations in alpha oscillations.^25^, ^26^

### 4.2 IAPF‐related BOLD responses

Regarding the IAPF‐related BOLD responses, we found BOLD signals in several subcortical and prefrontal areas fluctuated in phase with the time series of IAPF power fluctuations. The positive prefrontal correlates corroborate previous EEG studies demonstrating slowed alpha rhythms and their frontal spread in epilepsy.^23^, ^24^, ^25^ The positive correlates of thalamus and caudate corroborate previous BOLD findings of alpha oscillations in the brain.^35^, ^46^, ^47^ The positive cerebellar correlates appear to be in concordance with recent findings that patients with DRE exhibit cerebellar structural and functional alterations associated with specific gene expression patterns, suggesting a potential relevance to the mechanisms of drug resistance in DRE. In contrast to studies reporting negative BOLD correlates of power fluctuations in alpha oscillations,^35^, ^46^, ^47^ we speculated that the weakness of the effects might be a consequence of prolonged uncontrolled seizures in DRE.

Taken together, the overall IAPF‐related BOLD response patterns observed in our DRE patients differ from previous proposals of alpha oscillations and their role in cognitive control.^16^ This suggests that DRE may alter the functional role of alpha oscillations and reshape their cortical and subcortical representations. Notably, our findings closely align with a functional connectivity study that reported changes in thalamocortical connectivity in both focal and generalized epilepsy patients.^48^

### 4.3 Regulation effects of disease duration

In this study, epilepsy duration was correlated negatively with the IAPF in patients with DRE. By incorporating it as a covariate in fMRI data analysis, its modulation effects over the IAPF‐related BOLD response were revealed in several brain regions: (1) increased activation in the paracentral lobe and right insula; and (2) decreased activation in the bilateral orbitofrontal gyrus. These brain regions have been identified as part of central autonomic system, especially for sympathetic regulation.^49^, ^50^, ^51^, ^52^, ^53^ In addition, in a meta‐analysis of HRV and neuroimaging studies, the orbitofrontal gyrus, a subregion of medial prefrontal cortex, has been associated with autonomic and visceral aspects of emotional responses.^54^ By using IAPF as an instrument, our findings indicate that although epilepsy duration upregulates activities of alpha oscillations in the paracentral lobe and insula cortex, it downregulates the activities of alpha oscillations in orbitofrontal cortex. Our findings advance our understanding of the interaction between epilepsy and the central autonomic system by demonstrating distinct modulation effects over the IAPF‐related BOLD response in three regions of the central autonomic system.

## 5 LIMITATIONS

This study correlated relative power fluctuations of alpha oscillations with concurrent fMRI data, revealing several findings relevant to DRE, particularly in relation to the neural correlates of epilepsy duration. Although these findings are highly encouraging, they should be viewed in light of several considerations. First, the sample size of this study was moderate; expanding the cohort in future work would help further strengthen and consolidate the present results. Second, the boundaries between different oscillations within the 1–14 Hz range were not entirely clear. Although we targeted alpha oscillations ranging from 7 Hz to IAPF+2 Hz, it remains possible that oscillations below 7 Hz were also influenced by DRE. Third, alpha oscillations at rest arise from diverse anatomic sources. Because we focused on electrodes O1, O2, and Oz to calculate alpha power fluctuations, the precision of interpretation at the whole‐brain level may have been somewhat limited. Finally, there was no online monitoring of global spectral shifts, which may have made it more challenging to fully separate neural signals from those related to drowsiness or fluctuations in arousal.

## 6 CONCLUSION

Brain imaging plays a crucial role in various aspects of epilepsy assessment.^55^ For instance, structural imaging has revealed progressive hippocampal volume atrophy in patients with mesial temporal lobe epilepsy over time.^56^, ^57^ However, MRI findings in patients with DRE often appear normal.^58^, ^59^, ^60^ This study offers an alternative approach to identifying potential biomarkers associated with disease progression in patients with DRE.

## AUTHOR CONTRIBUTIONS

Jia‐Hong Sie had a role in conceptualization, formal analysis, investigation, methodology‐data analysis procedure development, visualization, and writing–original draft preparation. Hsin‐Ju Lee had a crucial role in methodology‐imaging protocol establishment and data curation. Yen‐Cheng Shih, Chien‐Chen Chou, and Chien Chen had crucial roles in data curation. David Niddam had a role in conceptualization and writing–review & editing. Hsiang‐Yu Yu had a role in conceptualization, resources, supervision, and writing–review & editing. Fa‐Hsuan Lin had a role in conceptualization, methodology‐imaging protocol establishment, and writing–review & editing. Wen‐Jui Kuo had a role in conceptualization, methodology, supervision, resources, writing–original draft preparation, and writing–review & editing.

## FUNDING INFORMATION

This work was supported by National Science and Technology Council, Taiwan (108‐2410‐H‐010‐006‐MY2, 109‐2314‐B‐075‐053, 112‐2314‐B‐075‐038‐MY2; 112‐2410‐H‐A49‐076‐MY2; 113‐2423‐H‐003‐004‐), National Health Research Institutes, Taiwan (NHRI‐EX109‐10905NI, NHRI‐EX112‐11229NI), Academia Sinica (AS‐ASSA‐113‐04).

## CONFLICT OF INTEREST STATEMENT

The authors declare no conflicts of interest.

## ETHICS STATEMENT

The study protocol was approved by the Taipei Veterans General Hospital Institutional Review Board, and the participants gave their written informed consent before scanning. We confirm that we have read the Journal's position on issues involved in ethical publication and affirm that this study is consistent with those guidelines.

## Data Availability

Anonymized data not published within this article will be made available upon reasonable request.
